# 

**Published:** 1910-11-26

**Authors:** 


					IMPORTANT TO HOSPITAL SECRETARIES.
ANALYSIS LEDGER.
Specially Ruled in accordance with, and adapted to the requirements of, the New Index of
Classification recently adopted by the King Edward's Hospital Fund for London,
and which is now in operation.
The Ledger is bound in half basil, green cloth sides, printed and ruled in best style on good paper, and is in every
way a serviceable book for Hospital use.
CABBIAGE EXTRA.
Secretaries requiring a copy of this Ledger should order at once to avoid delay.
TIHIE SCIEZESTTXEIC PRESS, LIMITED,
28 & 29 Southampton Street, Strand, London, W.C.
THE IDEAL WORK OF REFERENCE.
BURDETTS
HOSPITALS & CHARITIES.
1910 EDITION.
The Year-Book of Philanthropy and Hospital Annual.
Edited by
SIR HENRY BURDETT, K.C.B., K.C.V.O.
A Complete Guide and Directory of the Hospitals, Asylums,
and Charitable Institutions throughout the English-speaking
World.
Contains Special New Chapters on :?Voluntary Hospitals
in 1909.?Soundness of the Voluntary System.?The need of
greater zeal on the part of those who accept office.- State-
aided Hospitals in the U.S.A. and Canada.?The Appropria-
tion of Public Funds for the partial Support of Voluntary
Hospitals in U.S.A. and Canada.?The Need of an Apostle
and of greater interest on the part of the Clergy, &c. &o.
TWENTY-FIRST YEAR OF PUBLICATION.
Over 1,000 pages. Price 10/6 net; Post Free, 10/11.
OKLER your copy of the Medical Whitaker NOW.
Publishers :
THE SCIENTIFIC PRESS, Ltd., 28 & 29 Southampton St.,
Strand, London, W.C.
November 26, 1910. THE HOSPITAL
KEQAN PAUL, TRENCH, TRUBNER
& CO., LTD.
Sermon in the Hospital
BY MRS. HAMILTON KING.
F'cap 8vo. In leather 2/- net.
F'cap 8vo. In cloth 1 /-
Miniature Edition. In velvet calf . 1/6 net.
Miniature Edition. In leather . . 1/- net.
Within Hospital Walls
BY LADY LINDSAY.
Miniature Edition. In velvet calf . 1/6 net.
Miniature Edition. In leather . . 1/- net.
Readers of THE HOSPITAL are invited
to write to the Publishers for their Illus-
trated Catalogue of Autumn Books.
DRYDEN HOUSE, GERRARD ST., LONDON, W.
Just Issued. Ninth Edition, Rewritten and Enlarged, 9s. net.
PHARMACY, MATERIA MEDIGA & THERAPEUTICS.
Being a Commentary on the B.P. and its Drugs, with their Preparations,
and on all the new Non-official Remedies in use, with Chapters on the
different processes used in Dispensing, Prescription-writing, Latin
Glossary, &c. By Sir W. WHITLA, M.D., LL.D., Professor, Materia
Medica, Queen's University, Belfast, and Senior Physician, Royal Victoria
Hospital.
By the same Author. New Work. 1,900 pages. Orown8vo.25s.net.
PRACTICE OF MEDICINE.
Containing in two Handy Volumes a complete and independent Article on
the Etiology, Symptoms, and Diagnosis of every Medical Disease; arranged
in Dictionary Form for Practitioners and Students.
London: BAiixifeRE, Tindall <fc Cox, 8 Henrietta Street, 'W.C.
A MANUAL OF
MEDICAL EXERCISES.
By PERCY LEWIS, M.D., M.R.C.S.,
Hon. Medical Officer to Victoria Hospital, Folkestone.
66 pages, with 35 Illustrations, cloth, 13. 6d. net; post free, Is. 7d
H. K. LEWIS, 136 GOWER STREET, LONDON, W.C.
The FUTURE of the HOSPITALS.
Medical Relief for All according to means.
A Scheme of Co-operation between the PATIENTS, the
HOSPITALS, and the STATE.
By Sir HENRY BURDETT, K.C.B., K.C.V.O.
Price 2d.
THE SCIENTIFIC PRESS, LTD.,
28 & 29 Southampton Street, Strand, London, W.C.
And at all Bookstalls.
Every Medical Officer and all those engaged in Public
Health work, should read this booh.
A New and Simple Method of Treatment which renders Contagion impossible.
A PLEA FOR THE HOME TREATMENT
AND PREVENTION OF SCARLET FEVER
By ROBERT MILNE, M.D., Ch.M., Medical Officer of Dr. Barnardo's Hospitals and Homes.
In Cloth, 80 pp. 2S. post free.
The Lancet says :?" It is certainly desirable that steps should be taken to put the question of the true character of
scarlet fever on a certain basis. If it is really a mild disorder, which can be easily prevented from spread ng, then a large
number of beds in our isolation hospitals may be available for other purposes."
The Medical Times says :?" We may say definitely that we can, from our own personal experience, endorse every
word that Dr. Milne writes. We strongly advise our readers to study this book, and to act on the teaching therein contained.
It is a practical book which appeals to every medical man, as well as to those engaged in public health work. We wish
to give it our strongest recommendation."
The Hospital says:?" The method must stand or fall by its merits, and the issue of this book is welcome as
affording the profession an opportunity of hearing his claims and putting them to practical tests. Any medical officer toa
large institution of susceptible children should certainly give the plan a trial."
N.B.?Write for Nisbefs Complete List of Medical Publications, Post Free on Application.
Publishers: JAMES NISBET & CO., Ltd., 22 Berners Street, London, W.
Strangulated Hernia and Taxis.
The question of attempting to reduce a strangu-
lated hernia by taxis before proceeding to operation'
is a vexed one; much must depend on the circum-
stances of the individual case. In infants taxis will"
frequently prove successful, and an old-standing:,
large inguinal hernia, previously reducible, will
sometimes prove amenable, especially under an-
anaesthetic. But in a tightly nipped, recent hernia,,
especially of the congenital variety, it is almost
certain that taxis will not only be unsuccessful, but
will also do harm. Again, strangulated femoral1
herniee can far less often be reduced than inguinal-,
whilst umbilical herniae rarely yield. The probability
of failure, with the practical certainty of doing serious
damage to the bowel, justifies the general rule that
taxis is only to be used on the rarest occasions, and'
then only when the means of operation are at hand1,,
or when the patient is unsuitable even for an emer-
gency operation. Methods of local anaesthesia have
still further reduced the number of cases in which ii?
is justifiable to employ taxis.?Mr. Percy Sargent.
25S THE HOSPITAL November 26, 1910.
Obituary.
Mr. Mitchell Henry, formerly well known in Irish
politics, has died at the age of eighty-four. He was born
at Ardwick in 1826, and after being educated privately
and at University College, London, took his F.R.C.S. in
1854 and became surgeon to Middlesex Hospital. In
1862, however, he abandoned the medical profession to
become a partner in the mercantile firm of Messrs. A. and
S. Henry and Co., of Manchester and elsewhere; and in
1871 he entered Parliament as member for Galway County,
which he represented until 1885. In politics he was a
Home Ruler in the following of Isaac Butt. Mr. Henry
settled in his Irish constituency and built Kylemcre Castle,
between Letterfrack and Lenane, having purchased from
the Blakes a large estate of some 14,000 acres. The castle
itself was constructed at great cost and on land reclaimed
from bog; and Mr. Henry's residence and expenditure
brought abundant profit to the district. Some, few years
ago the property was purchased by the Duke of Man-
chester.
Dr. Joseph Frank Payne, M.D., F.R.C.P., Emeritus
Librarian to the Royal College of Physicians, consulting
physician to St. Thomas' Hospital, died at Lyonsdown
House, New Barnet, last week, in his seventy-first year.
Educated at University College, London, and Magdalen,
Oxford, he took a first in Natural Science in 1862, and the
next year became a Fellow of his College. The Burdett-
Coutts Scholarship in Geology, gained in 1863, and the
Radcliffe Travelling Scholarship in 1865, led to his taking
his B.Sc. at Oxford University; and in 1867 lie became
M.B. at Oxford. He studied at St. George's Hospital,
and, as a Travelling Scholar, at Paris, Vienna, and Berlin.
On his return Oxford overwhelmed him with appoint-
ments, and he became soon assistant physician at St.
Mary's and at the Hospital for Sick Children, Great
Ormond Street. In 1871 Dr. Payne began his thirty
years' connection with St. Thomas'. Lecturer there on
general pathology, materia medica, forensic medicine, the
principles and practice of medicine, Dr. Payne was simply
invaluable to the medical school and hospital alike. In
1873 he became F.R.C.P. and Gulstonian lecturer to the
College, where later the FitzPatrick and Lumleian lec-
tures were given by him. The College was indebted to
him also for his work on its behalf as examiner in
medicine and censor. In 1879 he accompanied Surgeon-
Major Colvill to investigate for the Russian Government
the causes of the plague. In 1892 he was appointed Sec-
retary to the Committee, presided over by Sir Andrew
Clark, for the preparation of a third edition of the
Nomenclature of Diseases. He had edited previously
Sieveking's " Manual of Pathology." In 1889 he be-
came Harveian Librarian, and entered joyfully on the
unfinished task of making a new catalogue. He gave
many rare volumes to the Library. He became a Fellow
of the Pathological Society in 1869, and in 1897 was made
its President. Of other societies we may note his pre-
sidency of the Epidemiological (twice) and of the Der-
matological, his vice-presidency of the Royal Society of
Medicine. Dr. Payne was a prolific writer in the journals
of learned societies and in literary and medical news-
papers. Apart from these miscellanea, his chief works
are the " Manual of Pathological Anatomy," " Observa-
tions on Some Rare Diseases of the Skin," the Life of
"Thomas Sydenham" ("Masters of Medicine Series"),
and fourteen lives in the "Dictionary of National Bio-
graphy."
The Consumption Crusade in Australia.?Tho build-
ing of the Consumption Hospital at the seaside, Chelten-
ham, is being proceeded with, as it is said the protest of
the inhabitants came too late, the contract being already
signed ! The hospital is within the grounds of a new
Benevolent Asylum now being built there, though a quarter
of a mile distant, and the two institutions are to be run in
conjunction in the matters of electric lighting, water sup-
ply, cooking, laundry work, and drainage on tho septic
tank system. Among Government projects for stamping
out tho disease is the prohibition of infected immigrants.
Incipient cases being somewhat difficult of detection, it is
considered that some power to deal with the affected
person after he has landed should be taken. The difficulty
of preventing consumptives leaving hospital or sanatorium
and going to live among the healthy is as great in Australia
as elsewhere, and it is probable that means of curtailing this
liberty and menace to public health will be created, at least
in Victoria.
26S THE HOSPITAL November 26, 1910.
Gifts and Bequests.
Lady Juliet Duff has received ?300 from Messrs. N.
M. Rothschild and Sons, and an anonymous ?100 in
response to her appeal on behalf of Charing Cross
Hospital. A promise of ?1,000 has also been received
from Lady Wantage.
Mr. Charles Todd, of Rodney Street, Liverpool, iron
and steel merchant, has bequeathed ?500 to the Children's
Infirmary, Liverpool.
Colonel Horace Montagu, of Pall Mall, S.W., late
8th and 11th Hussars, has left ?100 to the Trained
Nurses' Annuity Fund.
Prince Alexander of Teck has received ?500, which
was sent anonymously to the Queen, in addition to the
following donations, for the Prince Francis of Teck
Memorial Fund for the Middlesex Hospital : Mr. W. H.
Foster, ?50; Major-General and Mrs. J. B. B. D;ckson,
?20; Lord Stradbroke, ?10 10s.; Lady Blythswood, ?10.
At a meeting of the management committee of the Royal
?South Hants and Southampton Hospital last week it was
announced that Mies A. Burrell, of Fairthorn Manor,
Botley, Hants, had given ?7.000 towards providing a new
out-patients' building for the institution.
The Chelsea Hospital for Women, Fulham Road, has re-
ceived ?391, the bequest of the late Miss Isabella Blore.
Miss Hannah Hill, of Peterborough, has bequeathed
?2,000 each to the Peterborough Infirmary and to the
Hunstanton Convalescent Home. The residue of her
estate she left upon trust for a sister for life, with re-
mainder equally to the above-mentioned and other charit-
able institutions. The amount available for charitable
purposes under the will would appear to be about ?20,000.
Mr. James McLaren, of Portarlington Road, Bourne-
mouth, Hants, has bequeathed ?100 to Guy's Hospital.
Mr. James Tiiurlow, of London Road, High Wycombe,
corn merchant, has bequeathed ?200 upon trust for a charity
to be called the "Ellen Thurlow Charity," of which the
income is to be applied in gifts to each of twenty neces-
sitous and deserving persons resident in the parish of
Chipping Wycombe as may be selected by his trustees. Any
balance of income remaining after making these gifts is to
be given to the High Wycombe District Nursing Institu-
tion or some other charitable object in the discretion of
his trustees; ?20 each to the High Wycombo and Earl of
Beaconsfield Memorial Cottage Hospital; and ?10 to the
High Wycombe District Nursing Institution.
Mr. Alfred Lamb, of Durward House, Kensington
Court, W., left ?100 each to the Fresh x\ir Fund, Sir John
Kirk's Ragged School Union, the Kensington Dispensary,
and the Hospital for Children, Great Ormond Street, W.C.
Mr. Alexander Dishixgton, of Mottram Road, Staly-
bridge, Cheshire, more than thirty years manager of the
Manchester and Liverpool bank at Stalybridge, has left
?200 to the Northern Counties Hospital for Incurables,
stating that this bequest was paid in accordance with the
terms of his late wife's will.
Sir James Bailey, of Lofts Hall, Wenden, Saffron
Walden, Essex, and of 58 Rutland Gate, S.W., Conser-
vative member of Parliament for Walworth 1895-1906,
formerly a butler, has left ?100 each to the Saffron
Walden Hospital, the West London Hospital, Hammer-
smith, the Hospital for Women, Chelsea, the Hospital
for Diseases of the Chest, Fulham Road, S.W., and St.
George's Hospital, S.W., and ?50 to the Kensington Dis-
pensary and Children's Hospital.
Among the latest contributions acknowledged by the
Leicester Saturday Hospital Society are : from the em-
ployes of Messrs. Stead and Simpson, ?136 Is. 3d.; Messrs.
Moore, Eady and Murcott Goode, Ltd., ?121 9s. 9d.;
Messrs. W. Raven and Co., ?100; Messrs. S. D. Stretton
and Sons, ?92 5s. 8d. ; Leicester Co-operative Society's
employes, ?81; Messrs. Donisthorpe and Co., Ltd., ?60;
Ellistcwn Collieries, Ltd., workpeople, ?50; and the
trustees of Hanbury's Langton (Leicester) Charities have
sent a contribution of ?25 to the funds of the Leicester
Infirmary.
The Court of Common Council has voted ?52 10s. to
Queen Victoria's Jubilee Institute for Nurses.
Sir Edgar Speyer has given ?50, Messrs. NY M. Roth-
schild and Sons ?52 10s., and Mr. Ernest Riiffer l,212f. to
the Queen's Hospital for Children, Hackney Road, in
response to Lord Shaftesbury's appeal for ?8.500 to free
the institution from debt.
Mr. John Miller, of Richmond Hill, Clifton, solicitor,
has left ?100 to the Bristol Royal Infirmary, and ?50 each
to the Bristol General Hospital, the Bristol Eye Hospital,
and to the Royal Hospital for Sick Children and Women,
Bristol.
Mr. Thomas Wilkinson, of Bolton, has given ?1,000
towards the enlargement of Bolton Infirmary. The Gold-
smiths' Company has granted ?100 to Queen Charlotte's
Hospital, and ?250 to the general funds of University
College Hospital.
Miss Fanny Martyn, of St. Columb, Cornwall, after
making a number of private bequests, has left ?200 to
such parish in Cornwall as shall possess a crematorium for
the healthy and right means of disposing of the dead, and
if several parishes in Cornwall shall possess one, then to
such one as the trustees shall decide, and should no parish
in Cornwall have a crematorium, then this sum is to bo
paid to such parish as shall within twenty years of her
sister's death build a crematorium. The testatrix wished
to be cremated.
LARGE BEQUESTS TO CHARITIES.
Mrs. Sophia Letitia Barnard, of South Cave Castle,
Beverley, Yorks, who left estate of the gross value of
?125,469 Is. 5d., has bequeathed ?200 each to the Hull
Royal Infirmary and the Victoria Hospital for Sick
Children, Hull; and ?100 to the National Anti-Vivisection
Society. She directed her trustees, in their discretion,
within two years, to convey to any person or society or
corporation as they shall think fit such part of her real
estate not exceeding twenty acres as her trustees may
select for the purpose of the erection of a sanatorium or
other institution for the recuperation of health, if they
are satisfied that such person or body shall be able and
willing to provide funds for the erection and maintenance
of the said sanatorium, and in the event of such institution
being founded she empowered her trustees to apply a
sum of ?1.000 for the purposes of that institution as they
may think fit. To her servants she bequeathed a sum of
over ?900.
League of Mercy Meetings for the Week.?Monday,
November 28, Chelsea district : Mrs. Arthur Lyell at
home, 9 Cranley Gardens, S.W., 3.30 p.m. Tuesday,
November 29, Brixton district : Concert organised by the
lady Vice-Presidents, at Raleigh Hall, Effra Road, 8 p.m.
Appointments and Resignations.
Mr. John Wallace, M.A., M.B., Ch.B. Oxon,
M.R.C.S., L.R.C.P., has resigned the post of resident
medical officer at the Cardiff Infirmary after four years'
service.
Mr. R. Russell Thomas, M.R.C.S. Eng.. L.R.C.P.
Lond., of Cardiff, has been appointed honorary oph-
thalmic surgeon, and Mr. Norman B. Thornley, M.B.,
Ch.B., house surgeon to the special departments of the
Cardiff Infirmary. Dr. Thornley, who was educated at
Edinburgh University, was senior house surgeon for chil-
dren at Liverpool and house surgeon and house physician
at the Royal Southern, Liverpool. He was recently at St.
Paul's Eye and Ear Hospital, Liverpool.
H. H. Rayner, Esq., M.B., F.R.C.S. Eng.. has been
elected on the hon. medical staff of the Manchester
Children's Hospital as a " visiting surgeon."
Mr. L. Vernon Cargill, F.R.C.S., has been appointed
senior ophthalmic surgeon to King's College Hospital, in
?succession to Professor Malcolm McHardy, who has
resigned.
G. P. Putnam's Sons are publishing Dust and Its
Dangers, by Dr. Mitchell Prudden. This little work has
been written for the purpose of informing people, in simple
language, the real danger of serious disease?especially
consumption?in dust-laden air, and how this danger may
be avoided.
The death of Florence Nightingale, "the Lady of the
Lamp," has turned people's minds to her mission, and
made them ask, " Is that Lamp going out? " A reassur-
ing answer is given in the " History of Nursing," by Miss
Dock and Miss Nutting, published a short time ago by the
Putnams. It contains an interesting account of the be-
ginnings of nursing, with a full record of Miss Nightin-
gale's noble work in the Crimea, and goes on to describe
the great development of nursing in subsequent years.
November 26, 1910. THE HOSPITAL 273
Jottings.
Sib Herbert Tree has arranged to give a special
League of Mercy matinee at His Majesty's Theatre in the
first week of December. The bulk of the proceeds will be
devoted to the Prince Francis of Teclc Memorial Fund of
the Middlesex Hospital.
Founder's Day at the Royal Hospital for Incurables,
Putney Heath, will be celebrated at the hospital on.
Tuesday, November 29.
Princess Henry of Battenberg visited the Queen's
Hospital for Children at Hackney the other day, when a
general meeting of the Ladies' Association Work Guild was
held, under the presidency of Mr. Maurice Puffer
(treasurer).
The King has sent twenty brace of pheasants to the
West Norfolk and Lynn Hospital.
Over ?1,400 was made at a theatrical matinee in
Sydney, on September 5, for the purpose of providing the
Sydney Hospital with radium for treatment.
The opening of the new out-patient- department and the
*' Hulme" lift at the Poyal Southern Hospital, Caryl
Street, Liverpool, took place on Monday, November 21,
at 3 p.m.
Princess Louise (Duchess of Argyll), Princess Alex-
ander of Teck, Prince Alexander of Teck, and the Duke
of Argyll are patrons of a ball in aid of the Royal Waterloo
Hospital for Children and Women, to take place at the
Grafton Galleries on December 1, the birthday of Queen
Alexandra. Tickets may be obtained at the hospital,
Waterloo Road, S.E.
An appeal has just been issued from the members of
the Winchester Mission in Mandalay for a Children's
Hospital and convalescent home. The project has the
warm approval of the Bishop of Rangoon, as well as that
of the Winchester Diocesan Committee in connection with
the Mission, and will appeal to the sympathies of a wide
circle of friends. Donations should be sent to the Rev.
G. C. white, Nursling Rectory, Southampton, or to
Lloyds Bank Ltd., Southampton, for the Mandalay
Children's Hospital account.
As the result- of the special arrangements which Prince
Alexander of Teck has made for the convenience of those
who wish to contribute to the Prince Francis of Teck
Memorial I und by endowing a memorial bed or cot at
the Middlesex Hospital, a gentleman who wishes to re-
main anonymous has promised to forward 500 guineas
for the endowment of a cot on condition that four similar
offers are forthcoming by the end of this month.
The dance in aid of the Dreadnought Seamen's Hos-
pital, Greenwich, will take place on Tuesday, December 6,
in. Prince's Galleries, Piccadilly.
It is very much hoped that a cottage hospital will be
established at Brighouse, Yorkshire. Mr. W. K. MeGhie,
of Rastrick, has promised to give to the trustees enough
land for a site at Longrovd. Private generosity will
probably provide the necessary money for building.
Prince Alexander of Teck announces in reply to in-
quiries that arrangements have been made by which con-
tributors of 1,000 guineas to the fund may name a Prince
Francis Memorial bed in perpetuity. Contributors of 300
guineas will have a similar privilege during the life of the
donor, and contributors of thirty guineas for one year.
Plates with inscriptions recording the benefactions will be
placed over the beds.
Mr. John Burns opened the Park Hospital for Sick
Children, Hither Green, S.E., on Saturday, November 19,
at 2.30. We hope to give a full description of the adapted
hospital in an early issue.
A committee has been appointed to investigate tho
question whether a permanent medical officer at the Barry
Municipal Accident and Surgical Hospital, South Wales,
for whom at present no accommodation exists, would be
better than the present arrangements with the medical
men of the town. These arrangements expire at the end
of the year.
The annual festival dinner of the Poplar Hospital for
Accidents will be held at the Holborn Restaurant on
Wednesday, December 14, 1910, at 7 p.m. precisely. Sir
Owen Philipps, K.C.M.G., M.P., Vice-Chairman of the
Port of London, will be in the chair.
In connection with the Dolphin, Anchor, and Grateful
anniversary dinners held on November 13 last, when the
pious memory of the merchant and philanthropist, Edward
Colston, was paid tribute to in the form of considerable
collections for Bristol charities, it should be borne in mind
that the London medical fraternity, too, has occasion to
remember the beneficence of that great philanthropist. For
Edward Colston was liberal to London hospitals both in
his life-time and by the provisions of his will. He gave
?2,000 to St. Bartholomew's Hospital and ?1,000 to
Christ's Hospital, and subsequently bequeathed ?500 more
to the former and ?1,000 more to the latter, as well as
?500 to St. Thomas's Hospital and ?500 to Bethlehem
Hospital.
The following letter from Mr. A. William West,
treasurer and chairman of St. George's Hospital, appeared
in the Times of Tuesday last (November 22) : " Sir,?I
beg to state that not one penny of money subscribed to
St. George's Hospital for the benefit of the sick poor is
diverted for the purposes of school teaching. I am sending
this on my own responsibility, as there has not yet been
time to bring the subject of to-day's articles in the Press
before the House Committee of this hospital. I should be
much obliged if you could kindly insert this in to-morrow's
issue, as the articles that have appeared are likely to give
a false impression."
At a meeting convened by the Marquis of Winchester it
was decided that the Hampshire memorial to the late King
should take the form of a fund to be known as the " King
Edward Hospital Fund for Hampshire"; the object being
the raising and investing of as large a sum as possible,
the income from which should be devoted to the various
hospitals and nursing services of the county.
The working men and women supporters of the Coventry
and Warwickshire Hospital are to be congratulated on
having beaten even their previous record by having handed
over this month ?3,300 to the institution. This is ?400
more than was subscribed a year ago. The meeting at
which the money was presented took place in the new out-
patient department, which was built by the generosity of
the same working class.
Princess Victoria of Schleswig-Holstein presided
recently at the annual meeting of the Ladies' Association
of the Hampstead General Hospital, with which is amal-
gamated the North-West London Hospital. At the re-
quest of the Mayor of Hampstead (Alderman Ernest
Todd), her Highness unveiled a portrait of the late Mr.
W. F. Malcolm, who was hon. treasurer of the hospital
for twenty-eight years. The portrait was painted by Mr.
G. Hillyard Swinstead, R.I.
274 THE HOSPITAL November 26. 1910.
The Council of the Hospital Saturday Fund has decided
to close the Fund for the present year on Monday, January 9
next, at 6 p.m. The collections to Saturday last, according
to the latest weekly official statement, amounted to
?21,251 14s. 3d., being an increase as compared with the
corresponding period of last year of about ?3,000.
Prince Alexander of Teck presided at the court of
directors of the Royal Sea-Bathing Hospital on Monday,
when the Committee reported that the new ward and day
rooms were completed, and that there were now a few
vacancies for the male patients, but there were twenty
female applicants on the waiting list.
Countess Grosvenor opened the annual sale of work
of the ladies' committee of the Royal National Ortho-
paedic Hospital at the hospital, 234 Great Portland Street,
at half-past 2 o'clock on Thursday, November 24.
The committee appointed at Grosvenor House last
month to prepare a scheme for an Imperial memorial to
Miss Nightingale has held its first meeting at the India
Office in private.
Princess Louise (Duchess of Argyll) has given her
patronage to Dr. William A. Hall's annual concert, to be
held at the Municipal Hall, Tottenham, on December 8.
The proceeds, after defraying expenses, will be given to
the Prince of Wales's General Hospital, Tottenham.
A maternity hospital, to be known as the "King
Edward Memorial Hospital," is to be established at Perth,
the capital of Western Australia, the first of the kind in
that State.
The new Wagga Wagga (N.S.W.) District Hospital,
which is described as equal to the best outside of Sydney,
is provided with all modern comforts and antiseptic sur-
gical appliances, and has cost over ?12,000, was opened
on November 7 by the Premier of New South Wales (Mr.
Wade), who laid the foundation stone two and a half
years ago. Two days before, the patients were removed
in motor-car3 from the old building, over a mile away,
where a couple of cases of infectious diseases were left.
As everyone knows, a Coronation Exhibition is to be
held next year at Shepherd's Bush. Sine? hospitals have
figured very largely in the varied proposals for com-
memorating King Edward VII., it is interesting to note
that a sum of not less than ?5,000 of the receipts will
be devoted to ths Mansion House Fund for that purpose.
Mr. Halliwell Sutcliffe, the novelist, has written a
book about the work of the Royal Hospital for Incurables,
Putney Heath, which the governors of that institution
hope to publish at Christmas. Mr. Sutcliffe is a regular
visitor to the Hospital.
TO HOSPITAL SECRETARIES AND OTHERS.
We invite contributions from any of our readers, and
shall be glad to pay, according to length, for items of news
for the institutional section (including appointments, resig-
nations, gifts, etc.) which we may publish. All payments
for manuscripts used are made as early as possible after
the beginning of each quarter.
THE MEDICAL LIBRARY.
Post Free.
" Medical History: From the Earliest Times." By
E. T. Withington, M.A.. M.B. Oxon  12s. lOd.
" The Consumptive Working Man. What Can the
Sanatoria Do for Him ? " By Noel D. Bards-
well, M.D   - Is. 9d.
" Photomicrography." By Edmund Spitta,
M.R.C.S. With 41 Half-tone Reproductions
from original negatives and other Illustrations 12s. Od.
Of all booksellers or of The Scientific Press, Limited,
28 and 29 Southampton Street, Strand, London, W.C.
THE HOSPITAL November 26, 1910.
Name.
Address
This Coupon must accompany manuscript or contributions in-
tended for The Hospital.
A PRACTICAL GUIDE
TO
SURGICAL BANDAGING AND DRESSINGS.
A Pocket Book useful to the Student.
2s. post free.
MEDICAL HOMES FOR PRIVATE PATIENTS
A Classified Directory for the use of the Medical Profession.
Price 6d.
THE SCIENTIFIC PRESS, Ltd.,
28/29 Southampton Street, Strand, London, W.O.
INSTITUTIONAL WORKS.
HOSPITALS AND ASYLUMS OF THE WORLD.
By SIR HENRY BURDETT, K.C.B., K.C.V.O.
In Four Volumes, with Separate Portfolio containing several hundred Plans.
"Vols. I. and II. (ASYLUMS) ... Price ?6 15s. I Vols. III. and IV. (HOSPITALS)
and the Portfolio of Plans ... Price ?9
The Portfolio Of Plans (20 in. x 14 in., drawn to uniform scale), Price ?4 14 6
The Complete Work   ?12 12s.
This magnificent work forms a Complete Survey of the Origin, History, Construction, Administration and Management
of the Hospitals and Asylums of the World, with detailed information concerning the principal Institutions.
Only a few copies remain unsold.
LEDGERS, ACCOUNT BOOKS, STOCK BOOKS of all kinds for large
and small Institutions.
These are supplied already ruled and printed, or in accordance with the special requirements of institution managers.
THE SCIENTIFIC PRESS, LTD.,
28 and 29 Southampton Street, Strand, London, W.C.

				

